# Automatic diagnosis of strict left bundle branch block using a wavelet-based approach

**DOI:** 10.1371/journal.pone.0212971

**Published:** 2019-02-25

**Authors:** Alba Martín-Yebra, Juan Pablo Martínez

**Affiliations:** 1 Centro de Investigación Biomédica en Red—Bioingeniería, Biomateriales y Nanomedicina, Universidad de Zaragoza, Zaragoza, Spain; 2 BSICoS Group, Aragón Institute of Engineering Research, IIS Aragón, Universidad de Zaragoza, Zaragoza, Spain; University of the Basque Country, SPAIN

## Abstract

Patients with left bundle branch block (LBBB) are known to have a good clinical response to cardiac resynchronization therapy. However, the high number of false positive diagnosis obtained with the conventional LBBB criteria limits the effectiveness of this therapy, which has yielded to the definition of new stricter criteria. They require prolonged QRS duration, a QS or rS pattern in the QRS complexes at leads V1 and V2 and the presence of mid-QRS notch/slurs in 2 leads within V1, V2, V5, V6, I and aVL. The aim of this work was to develop and assess a fully-automatic algorithm for strict LBBB diagnosis based on the wavelet transform. Twelve-lead, high-resolution, 10-second ECGs from 602 patients enrolled in the MADIT-CRT trial were available. Data were labelled for strict LBBB by 2 independent experts and divided into training (n = 300) and validation sets (n = 302) for assessing algorithm performance. After QRS detection, a wavelet-based delineator was used to detect individual QRS waves (*Q*, *R*, *S*), QRS onsets and ends, and to identify the morphological QRS pattern on each standard lead. Then, multilead QRS boundaries were defined in order to compute the global QRS duration. Finally, an automatic algorithm for notch/slur detection within the QRS complex was applied based on the same wavelet approach used for delineation. In the validation set, LBBB was diagnosed with a sensitivity and specificity of Se = 92.9% and Sp = 65.1% (Acc = 79.5%, PPV = 74% and NPV = 89.6%). The results confirmed that diagnosis of strict LBBB can be done based on a fully automatic extraction of temporal and morphological QRS features. However, it became evident that consensus in the definition of QRS duration as well as notch and slurs definitions is necessary in order to guarantee accurate and repeatable diagnosis of complete LBBB.

## Introduction

Left bundle branch block (LBBB) consists of a blockage in the propagation of the electrical impulse through the main left branch. As a consequence, activation of left ventricular wall is delayed which respect to the interventricular septum, leading to an inefficient pumping of the heart (heart failure). Cardiac resynchronization therapy (CRT) has been postulated as the preferred option for resynchronization of ventricular contraction in heart failure patients with reduced ejection fraction [1]. Among them, patients with LBBB have been shown to have better clinical response to CRT [2,3].

Conventional diagnosis is based on electrocardiographic (ECG) criteria, typically requiring a prolonged QRS duration (≥ 120 ms) and QS or rS configurations in lead V1 [4]. However, approximately one third of diagnosed patients were shown not to have complete LBBB [5,6], revealing that there is a lack of a gold standard for true LBBB diagnosis. Indeed, it is the high false-positive rate obtained with conventional LBBB criteria what mainly limits the effectiveness of CRT for treating those heart failure patients [5,7].

Later on, differences in QRS duration between men and women with LBBB were found, and simulation studies evidenced the presence of mid-QRS notches or slurs in some leads when complete LBBB was present. These observations yielded to the definition of new stricter criteria for LBBB diagnosis [7]. They require three simultaneous conditions: C1) prolonged QRS duration (≥140 ms in men, ≥130 ms in women), C2) QS or rS pattern in the QRS complexes at leads V1 and V2 and C3) the presence of mid-QRS notches/slurs in ≥2 of leads within V1, V2, V5, V6, I and aVL.

In 2018, the International Society for Computerized Electrocardiology (ISCE) and the Telemetric and Holter Warehouse (THEW) project prompted the LBBB initiative in order to give research teams the opportunity to test automatic algorithms for strict LBBB diagnosis in patients with heart failure and reduced ejection fraction from the MADIR-CRT trial [8]. The aim of this work, as part of the LBBB initiative, is to develop and assess a fully automatic algorithm for strict LBBB diagnosis using a wavelet-based approach.

## Materials and methods

### Study population

Data available in this initiative are part of the Multicenter Automatic Defibrillator Implantation Trial—Cardiac Resynchronization Therapy (MADIT-CRT), conducted at University of Rochester (Rochester, NY) [9]. The original study aimed to investigate whether CRT would reduce mortality and heart failure events in patients at mild heart failure stages. The original dataset included 1820 randomized patients in New York Heart Association classes I and II, 1281 with LBBB (diagnosed according to conventional criteria), from 110 different hospital centers. Twelve-lead, high-resolution ECGs (sampling frequency of 1000 Hz and amplitude resolution 3.75 μV) were recorded before CRT implantation using 24-hours Holter recorders (H12+, Mortara Instruments, Milwaukee, WI, USA) during 20 minutes in supine position. The study protocol was approved by each institutional review board of the participating centers.

For the present study, the organizers of the LBBB initiative only provided ECGs and gender labels from a subset of the MADIT-CRT patients. A total of 602 10- second ECG traces in sinus rhythm as well as the median beat for each of the 12 standard leads were made available to the participants. The dataset (72% men, 28% women) was divided by organizers into two sub-cohorts, conforming the training (n = 300 recordings) and validation datasets (n = 302 recordings, with diagnostic annotations blinded to the investigators before submission). Reference annotations such as global QRS duration, QRS configurations of leads V1 and V2 and the presence of notches/slurs were delivered only for the training dataset. Data were labelled for strict LBBB by 2 independent experts, with an additional third reviewer involved if tie-break consensus was needed. No other clinical information from the trial was available.

According to the strict LBBB criteria, diagnosis of LBBB requires delineation of QRS boundaries, identification of QRS morphological pattern as well as the presence of notches and/or slurs within the QRS complex in selected leads. The methodology proposed here is based on the same wavelet approach used in [10] for ECG delineation.

### Multilead QRS delineation

First, individual *Q*, *R* and *S* waves as well as QRS boundaries (*QRS*_*on*_ and *QRS*_*off*_) were detected on median beats for each standard lead using a wavelet-based delineator. We refer the reader to [10] for a detailed description of the algorithm and its implementation. Briefly, this ECG delineator uses the derivative of a smoothing function as a prototype wavelet. Therefore, the obtained coefficients can be identified as the derivative of the low-pass filtered signal at different scales, acting as a differentiator filter-bank.

In this wavelet decomposition of the ECG signal, the most significant components of the ECG are contained in scales *k* = {1,…,4}. In particular, QRS peaks correspond to zero-crossings at scale 1 between pairs of maximum moduli with opposite sign at scale 2. Negative deflections, such as *Q* and *S* waves, appear between a negative minimum-positive maximum pair, whereas positive deflections, such as *R* or *R*^*’*^ waves, are identified between a positive maximum-negative minimum pair at scale 2. The original algorithm included some rules and protections based on time and sign conditions to reject deflections not defining a QRS wave (such as notches/slurs or noise artifacts).

To identify QRS boundaries, the first step was the identification of the first and last significant slopes of the QRS complex, which correspond to peaks of the wavelet transform at scale 2. The *QRS*_*off*_ mark was set as the first sample after the last slope of the QRS where the wavelet transform at scale 2 falls below a given threshold. The *QRS*_*on*_ was defined in a similar way considering the first slope of the QRS complex.

**Fig 1:**
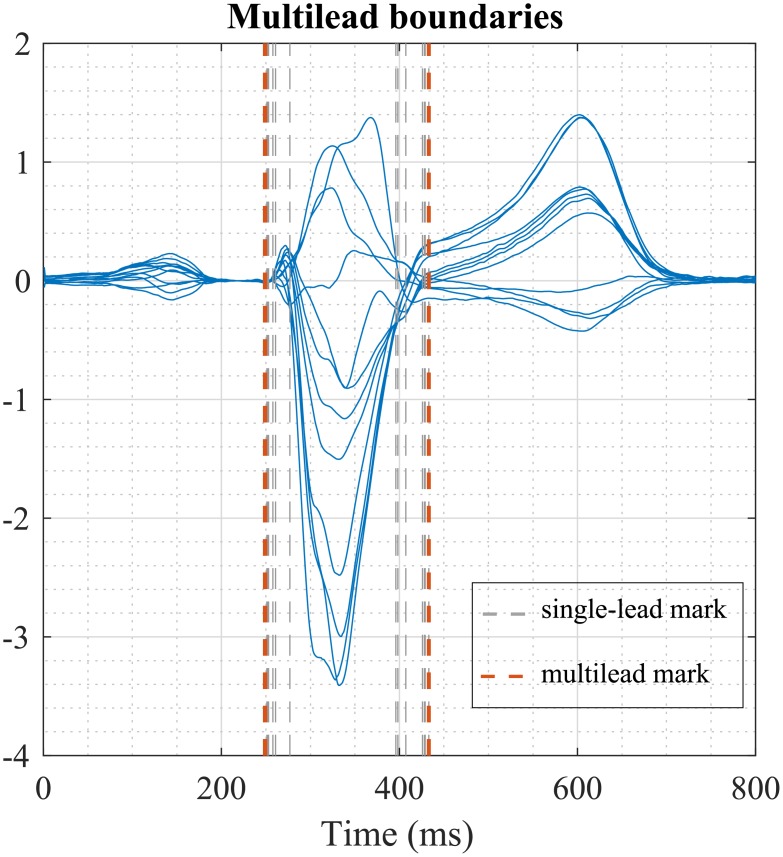
Multilead QRS boundaries (red dashed lines) obtained by applying post-processing selection rules over the single-lead marks (grey dashed lines).

From those single-lead annotations, multilead QRS boundaries were defined. This multilead detection was based on post-processing selection rules applied over all single-lead annotations, thus providing a more robust delineation [10]. Post-processing rules for boundaries consisted of ordering all 12 single-lead marks and setting the onset of the QRS complex (${QRS}_{on}^{multi}$) as the earliest mark with at least *m* = 2 nearest neighbours within a *δ* = 10 ms interval. In the same way, the end of the QRS complex (${QRS}_{off}^{multi}$) was set as the latest annotation mark with *m* = 2 neighbour marks in a *δ* ms interval (Fig 1). Finally, global QRS duration (*QRSd*) was computed as the difference between ${QRS}_{off}^{multi}$ and ${QRS}_{on}^{multi}$ positions.

### QRS morphological patterns

The second condition for LBBB diagnosis requires QS or rS configurations (Fig 1) in leads V1 and V2, in contrast to normal left-to-right activation of the septum, which is associated with the presence of a *R* wave in those leads.

After association of the global QRS position to the most prominent deflection within the QRS complex, the wavelet-based algorithm searches for all individual *Q*, *R*, *S* or R^*’*^ waves, considering any possible QRS morphological configuration (i.e, QRS, RSR’, RS, R, QR or QS) [10].

- A **QS configuration** requires that only a negative deflection is detected, identified as the main wave of the complex, which corresponds to the *S* wave. No *R* wave is detected.
- An **rS configuration** also requires the main wave to be a negative deflection, corresponding to the *S* wave, but in this case preceded by a positive deflection of lower amplitude respect to the isoelectric line, denoted as the *r* wave. No *R*^*’*^ wave is detected after the *S* wave.

### Notch/Slur detection

Finally, using the same wavelet-based multiscale approach as for ECG delineation, an algorithm for notch/slur detection was developed. As it has been mentioned before, any peak/nadir on the ECG signal corresponds to a zero-crossing at scale 1. We denoted those zero-crossings as *z*_*i*_, i = {1,…,*I*}, with *I* the total number of detected zeros at this scale.

A notch present on the QRS complex is defined by three consecutive zero-crossings at scale 1 having the same polarity on the ECG signal, that is:

$$sign(x_{ECG}(z_{i-1}))\;=\;sign(x_{ECG}(z_{i}))\;=\;sign(x_{ECG}(z_{i+1}))$$ (1)

where *x*_*ECG*_ denotes the ECG signal and *z*_*i*-1_, *z*_*i*_ and *z*_*i*+1_ are the consecutive zero-crossings at scale 1. Notch boundaries correspond to the first and the last zero of the triplet, respectively.

A slur appears as a notch on the first scale of the wavelet transform (*x*_*WT*1_). Thus, the algorithm uses the same strategy but applied to this signal and its derivative. In this case, after detection of all zero-crossings (${z'}_{j}$, j = {1,…,*J*}) on the derivative of *x*_*WT*1_(*n*) within the QRS complex (${x^{'}}_{WT1}(n)$), slur boundaries correspond to the positions of ${z'}_{j-1}$ and ${z'}_{j+1}$ for any *j* that fulfills the following:

$$sign(x_{WT1}({z'}_{i-1}))\;=\;sign(x_{WT1}({z'}_{i}))\;=\;sign(x_{WT1}({z'}_{i+1}))$$ (2)

**Fig 2:**
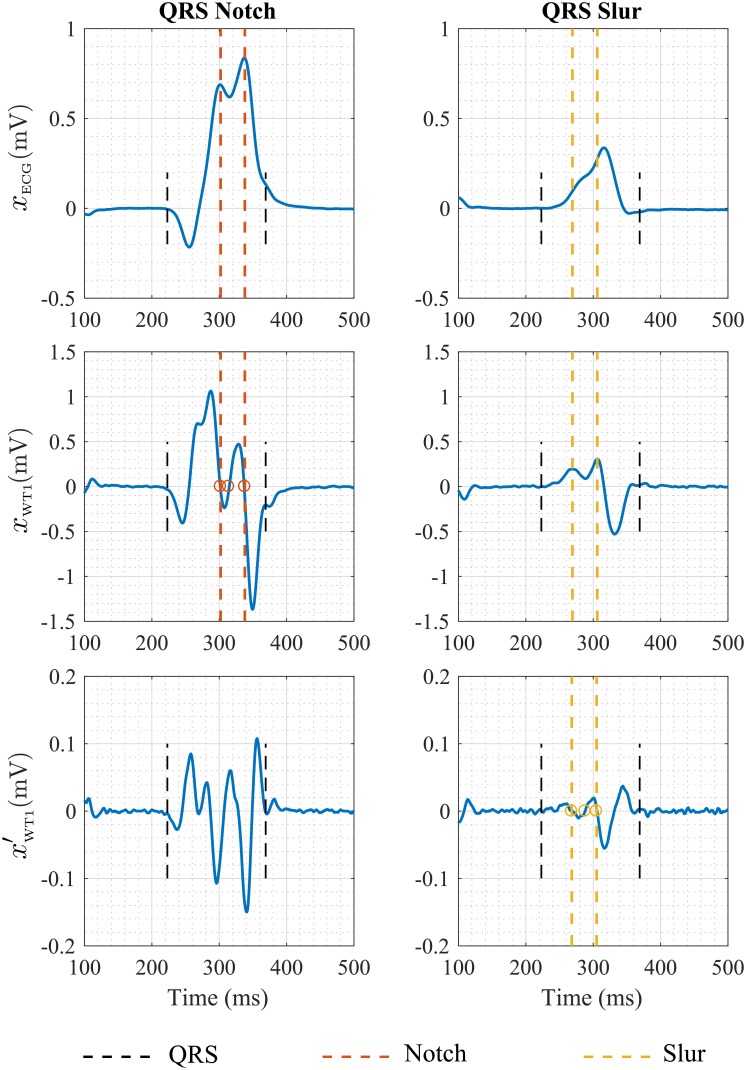
Two QRS complexes with a mid-QRS notch (left) and slur (right), respectively. The corresponding wavelet-transform signal at scale 1 and its derivative are also shown in order to illustrate the definition of notch (red) and slur (yellow) boundaries from consecutive zero-crossings (o).

Illustrations of both notch (left) and slur (right) detection are shown on Fig 2. According to the aforementioned strict LBBB criteria, mid-QRS notches/slurs need to appear 40 ms after the QRS onset and before 50% of the global QRS duration to be associated to LBBB. In addition, they are required to be present in at least 2 of leads V1, V2, V5, V6, I and aVL. Initially, strict LBBB criteria also included an additional condition, requiring the notch/slur to end before 2/3 of the QRS duration. However, after *ISCE 2018* annual meeting this condition was discarded.

### Parameter adjustment

At the time of submission, gender label, QRS boundaries and duration, the presence of QS o rS configurations in V1 and V2 as well as notch/slur annotations in selected leads were made available only for the training set. However, only sex information was provided for the validation set. We used the training set (n = 300) for learning purposes. In particular, it was used to adjust some parameters of the wavelet-based delineator for the particular condition of prolonged QRS complexes and the determination of QRS and notch/slurs boundaries. Afterwards, the method was blindly applied to the test set (n = 302) to obtain the results submitted to the Initiative. After ISCE2018, the corrected reference annotations and adjudications from both training and validation datasets were public to all participants. The methodology and results presented in this work only differed from the submitted ones in that we excluded the condition for the notch/slur end to end before 2/3 of the QRS duration, according to the changes to the criteria made by the organizers.

## Results

We used the manual annotations from the training dataset for the adjustment of several parameters of the ECG delineator. This cohort included a total of 174 strict LBBB cases, and final diagnosis was obtained with an accuracy rate of Acc = 83.7% (Se = 93.7%, Sp = 69.8%) using the automatic algorithm. Results from the validation set are included on Table 1. As annotations were public after ISCE2018 meeting, in addition to final LBBB diagnosis we have evaluated the individual performance of C1 and C2 conditions. Results are included on Table 1.

**Table 1:** Performance of the algorithm for automatic LBBB diagnosis on the validation dataset (n = 302 ECGs, 156 LBBB cases) with respect to the manually annotated references. Individual performance of conditions C1 (QRS duration ≥130ms in woman and ≥140ms in men), and C2 (rS or QS configurations in leads V1 and V2) is also included.

	Acc (%)	Se (%)	Sp (%)	PPV (%)	NPV (%)
LBBB diagnosis	79.5	92.9	65.1	74.0	89.6
C1 (*QRSd*)	82.1	97.3	42.2	81.9	85.4
C2 (QRS conf)	96.0	98.3	86.9	96.7	93.0

Acc: Accuracy; Se: Sensitivity; Sp: Specificity; PPV: Positive predictive value; NPV: negative predictive value; LBBB: Left bundle brunch block; *QRSd*: QRS duration; QRS con: QRS complex configuration.

**Fig 3:**
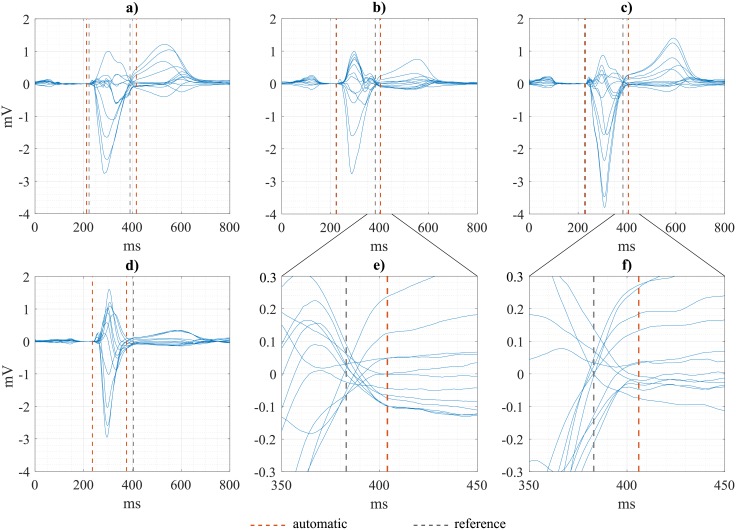
Illustration of automatic (red dashed lines) and reference annotations (grey dashed lines) of multilead QRS boundaries in some ECGs. Panels e) and f) show a detailed view of the end part of the QRS of examples b) and c).

Using the multilead boundaries from the ECG delineator, we got wider *QRSd* values than with manual annotations (absolute difference: ε = 10.1 ± 14.8 ms). This led to a significant number of false positives with respect to the experts’ adjudications, which explains the low specificity rate (42.2%) obtained when evaluating the C1 condition. Examples of some of the biggest discrepancies between manual (grey) and automatic (red) annotations are shown in Fig 3.

**Fig 4:**
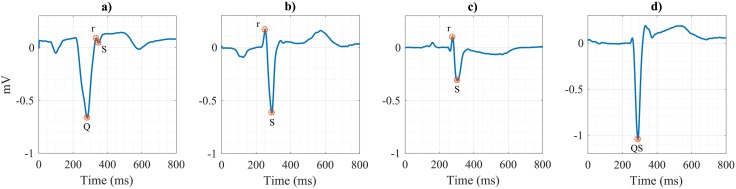
Different QRS configurations identified by automatic annotations obtained from the ECG delineator. Panel a) illustrates a false negative QrS configuration, while panels b), c) and d) illustrate 3 false positive rS, rS and QS configurations, respectively, according to the reference annotations.

On the other hand, from the automatic *Q*,*R*,*S*peaks detections, QRS morphological configurations in leads V1 and V2 were accurately identified (Acc = 96.0%). Only 12 out of 302 cases were misclassified (8 false positives and 4 false negatives) with respect to the provided annotations. Some of them are illustrated on Fig 4.

**Fig 5:**
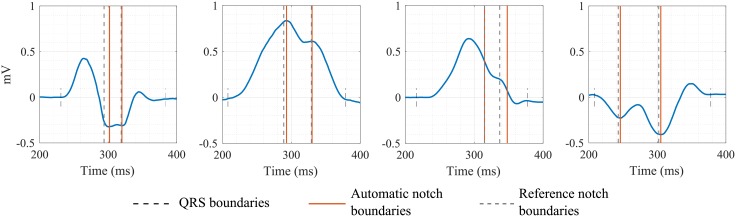
Examples of automatic (red lines) and reference (grey dashed lines) boundaries of mid-QRS notches/slurs of some QRS complexes.

Disagreements in C3, i.e., the presence of mid-QRS notches/slurs between the automatic algorithm and reference annotations could not be quantified since experts’ annotations of notches/slurs were not provided when one of the two previous conditions (C1 or C2) were not fulfilled. Also, no more than 3 slurs or notches were annotated in a QRS complex. We show some examples of mid-QRS notch/slur detections in Fig 5.

Altogether, taking manual annotations as a gold standard, diagnosis of strict LBBB in the test dataset (156 cases out of 302 ECGs) was achieved with an accuracy of 79.5% and a sensitivity and specificity rates of Se = 92.9% and Sp = 65.1% in a fully automatic way. From the total cohort, we identified 62 ECGs that were differently classified by the manual annotations and the algorithm. The origin of the low Sp was the significant number of false positive cases (51), 38 of them due to the *QRSd* overestimation (disagreements on C1).

## Discussion

This work was part of the LBBB initiative, prompted on the 43^rd^ ISCE meeting. Using the same wavelet approach implemented in our ECG delineator [10], we have developed a methodology for fully-automatic diagnosis of strict LBBB. After identification of main *Q*, *R*, *S* waves and QRS boundaries, the algorithm takes the advantage of the wavelet transformation for the detection and delineation of mid notches/slurs, adding additional capabilities to the original delineator.

The strict LBBB criteria proposed by Strauss et al. [7] arise as a consequence of the reduced effectiveness of CRT therapy in patients diagnosed with conventional LBBB but actually did no present a complete blockage on the left bundle. These criteria are defined by three simultaneous conditions based on temporal and morphological features of the QRS complex.

The first condition (C1), as for conventional criteria, requires a prolonged QRS complex, but in contrast to the ≥120 ms threshold, a gender-dependent threshold was imposed. We applied multilead post-processing rules over the 12 single-lead annotations given by the ECG delineator for the definition of QRS boundaries and the computation of global QRS duration. We observed an overestimation of this measurement of 10 ms on average when compared to the experts’ annotations. We identified that these differences were mainly due to a delayed position of the ${QRS}_{off}^{multi}$ mark respect to manual annotations, obtained from a butterfly plot. As a result, more than a half of misdiagnosed LBBB (38 out of 62) were attributable to disagreements on this condition. However, from the examples shown on Fig 3, it can be observed that the change of the slope determining the end of the depolarization phase (end of the QRS complex) is better approximated by the automatic (red) measurement than by the reference one, obtained from a butterfly plot. In the later, the point of minimal variation between leads tends to be the one selected as ${QRS}_{off}^{multi}$. It is evident from the examples that it exists a lack of consensus in the definition of global QRS limits and, therefore, in the computation of QRS duration. This becomes critical to guarantee the reproducibility of the measurements and, more importantly, for accurate diagnosis to guide therapy decisions.

The second condition is related to the QRS morphology of leads V1 and V2. Highly accurate results were obtained in the identification of QS and rS configurations from automatic *Q*, *R*, *S* annotations of those leads (Acc = 96%). Main discrepancies (6 out of 8 false positives) were due to an rS decision, while from experts’ annotations they were categorized as RS, therefore not accomplishing C2. Also in this case, it seemed that there is not a general agreement for the decision of a significant (*R*) or small (*r*) wave. In our algorithm, we required the absolute amplitude of *R* wave respect to the isoelectric line to be lower than 2/3 of the *S* wave amplitude, (i.e, R/S <2/3).

The third and last condition imposes the presence of mid-QRS notches or slurs in some leads. For this purpose, we have used the same approach as for delineation of main ECG waves based on a wavelet transformation [10]. Originally, the delineation method included some temporal- and amplitude-based protections in order to ignore any signal deflection associated to noise artifacts, notches or slurs. In this work, we have developed a new module for the detection and location of these notches/slurs. Notches/slurs were only manually annotated if the two first conditions were positive. Moreover, there were never more than 3 annotated notches o slurs, which limited the possibility of assessing the performance of our algorithm for notch/slur detection. Nevertheless, only 19 out of 62 final inaccurate LBBB diagnosis were due to discrepancies in this third condition. For the cases where annotations were available, we observed some discrepancies in the delimitation of notch/slur boundaries between our automatic annotations and the reference annotations. As it happened for QRS duration, there is not a clear definition for how long or how deep a notch/slur must be to be considered as such. While there is a general consensus in the definition of a slur as a notch on the derivative signal, different temporal and amplitude thresholds have been used to consider the notch significant [11,12].

Results of the LBBB initiative according to the uncorrected strict LBBB criteria (including the restriction to the end of the notch/slur) have been recently published in [8]. With those criteria, we obtained an accuracy of 71% on test dataset (5^th^ position). The best accuracy attained among all participants with respect to the provided annotations was 82% and detailed implementation of its methodology was presented in [11]. Nonetheless, it becomes evident from this work that a prior consensus in the definition of main electrocardiographic intervals and features, as global QRS duration or notches, is extremely necessary for reliable and repeatable diagnosis based on these criteria. On the other hand, the definition of strict LBBB was changed even during the initiative, suggesting that the debate about what would be the best definition is still open. In addition to the strict LBBB criteria evaluated in this work, the European Society of Cardiology (ESC) and the American Heart Association (AHA) have their own definitions, with slightly different criteria among them [13–16]. Those differences mainly lie in a different threshold for *QRSd*, the assessment of discordant T waves or negative T waves in leads with upright QRS. Although our algorithm has been adapted to evaluate the strict LBBB criteria it can be easily adapted to the other LBBB definitions.

From the clinical point of view, one major limitation of this study was the unavailability of the final CRT outcome in selected patients, making it impossible to test the actual clinical impact of the method in this cohort. However, the LBBB initiative was purely technically-oriented and organizers only provided the strictly necessary data for diagnosis. Furthermore, it has been recently shown that CRT outcome is significantly influenced by the LBBB definition [13]. Indeed, there is a lack of an ECG-based standard definition for LBBB. As we have mentioned before, the ESC and the AHA have published their own guidelines, which have been continuously adapted along the last decade. Among all definitions, the strict LBBB criteria together with the ESC 2009 and ESC 2013 are the only ones that significantly stratify patients according to CRT outcome and heart failure hospitalizations [13].

## Conclusions

In conclusion, the presented methodology allows diagnosis of strict LBBB based on a fully automatic extraction of temporal and morphological QRS features. However, consensus in the definition of QRS duration as well as notch and slur definitions becomes necessary in order to guarantee a proper and repeatable diagnosis of strict LBBB based on precise and reliable measurements.
